# Regulation of PUMA induced by mechanical stress in rat cardiomyocytes

**DOI:** 10.1186/1423-0127-19-72

**Published:** 2012-08-03

**Authors:** Wen-Pin Cheng, Gong-Jhe Wu, Bao-Wei Wang, Kou-Gi Shyu

**Affiliations:** 1 Department of Medical Education and Research, Shin Kong Wu Ho-Su Memorial Hospital, Taipei, Taiwan; 2 Department of Anesthesiology, Shin Kong Wu Ho-Su Memorial Hospital, Taipei, Taiwan; 3 School of Medicine, College of Medicine, Taipei Medical University, Taipei, Taiwan; 4 Division of Cardiology, Shin Kong Wu Ho-Su Memorial Hospital, 95 Wen-Chang Rd, Taipei, 111, Taiwan; 5 Graduate Institute of Clinical Medicine, College of Medicine, Taipei Medical University, Taipei, Taiwan

**Keywords:** Cardiomyocytes, PUMA, Volume overload, Cyclic stretch, Atorvastatin

## Abstract

**Background:**

PUMA (p53-up-regulated modulator of apoptosis), an apoptosis regulated gene, increased during endoplasmic reticulum stress. However, the expression of PUMA in cardiomyocytes under mechanical stress is little known. We aimed to investigate the regulation mechanism of PUMA expression and apoptosis induced by mechanical stress in cardiomyocytes.

**Methods:**

Aorta-caval (AV) shunt was performed in adult Wistar rats to induce volume overload. Rat neonatal cardiomyocytes were stretched by vacuum to 20% of maximum elongation at 60 cycles/min.

**Results:**

PUMA protein and mRNA were up-regulated in the shunt group as compared with sham group. The increased PUMA protein expression and apoptosis induced by shunt was reversed by treatment with atorvastatin at 30 mg/kg/ day orally for 7 days. TUNEL assay showed that treatment with atorvastatin inhibited the apoptosis induced by volume overload. Cyclic stretch significantly enhanced PUMA protein and gene expression. Addition of c-jun N-terminal kinase (JNK) inhibitor SP600125, JNK small interfering RNA (siRNA) and interferon-γ (INF-γ) antibody 30 min before stretch reduced the induction of PUMA protein. Gel shift assay demonstrated that stretch increased the DNA binding activity of interferon regulatory factor-1. Stretch increased, while PUMA-Mut plasmid, SP600125 and INF-γ antibody abolished the PUMA promoter activity induced by stretch. PUMA mediated apoptosis induced by stretch was reversed by PUMA siRNA and atorvastatin.

**Conclusions:**

Mechanical stress enhanced apoptosis and PUMA expression in cardiomyocytes. Treatment with atorvastatin reversed both PUMA expression and apoptosis induced by mechanical stress in cardiomyocytes.

## Background

Hypertrophy, a major determinant of morbidity in all forms of cardiovascular diseases, has become the increasing cardiovascular disorder in the developed countries [1]. Hypertrophic heart often leads to dilated cardiomyopathy, and finally leads to congestive heart failure after sustained overload [2]. Thus, understanding the molecular mechanisms and developing novel therapeutic agents for patients with hypertrophy remains a major research priority. Cardiac hypertrophy, which occurs in response to increased mechanical load on the heart in the form of pressure or volume overload, is characterized by increased cell size, enhanced protein synthesis, and re-expression of fetal genes.

The etiology of heart failure involves multiple agents and conditions, but the progressive loss of cardiomyocytes is one of the most important pathogenic components. Cardiac apoptosis may be an important factor during the transition from compensatory hypertrophy to heart failure [3]. The role of cardiomyocytes apoptosis during heart failure is not completely understood. Therefore, the possibility of reducing cardiomyocytes loss by inhibiting apoptosis has potentially important implications in the treatment of heart failure. To prevent the progression of hypertrophy and heart failure, it is important to have a full understanding of the apoptosis mechanism in cardiomyocytes and thus to identify potential therapeutic targets [4].

Apoptosis pathways include death initiated by ligation of membrane-bound death receptor, release of proapoptotic factors from mitochondria or stress at the endoplasmic reticulum (ER). ER is a central organelle entrusted with lipid synthesis, calcium homeostasis, protein folding, and maturation [5]. Thus, the development of the ER in hypertrophic and failing hearts may implicate the compensatory response to the up-regulated protein synthesis. Correctly folded proteins exit the ER and are transported to the Golgi and other destinations within the cell, but proteins that fail to fold properly misfolded proteins are retained in the ER and their accumulation may constitute a form of stress to the cell called ER stress [6]. In the heart, hypoxia, ischemia/reperfusion, hypertrophy, pressure overload, and drug-induced insults can result in activation of ER stress [7]. In order to overcome ER stress and maintain cell homeostasis, ER stress triggers a specific signaling pathway called unfolded protein response (UPR). As ER stress is excess and/or prolonged, the UPR can not reverse the damage and then causes the cell to undergo apoptosis [8].

One component of the ER stress-mediated apoptosis pathway is the p53-upregulated modulator of apoptosis (PUMA), also known as Bcl-2 binding component 3 [9,10]. PUMA is a Bcl-2 homology 3 (BH3)-only Bcl-2 family member and a critical mediator of p53-dependent and -independent apoptosis under various stimuli in several tissues and cells [11,12]. Besides, interferon regulator factor-1 (IRF-1) could bind to the promoter of PUMA and activate PUMA transcription induced by interferon-γ [13].

PUMA has been induced by cyclic stretch in VSMCs [14]. This system has been applied widely in studying the molecular mechanisms of gene expression and signal transduction in many cell types [15]. There was a good evidence to show that PUMA plays an important role in cardiomyocytes apoptosis upon ER stress and ischemia/reperfusion [16]. However, there is no conclusive proof on how mechanical cyclic stretch affects the PUMA on the apoptosis in cardiomyocytes.

The 3-hydroxy-3-methylglutaryl coenzyme A (HMGCoA) reductase inhibitors, statins, have been associated with reduced morbidity and mortality in patients with coronary artery disease [17]. The application of atorvastatin in patients with non-ischemic heart failure improved left ventricular ejection fraction and attenuated adverse left ventricular remodeling [18]. The beneficial effects of atorvastatin in attenuating cardiac hypertrophy also extend to acquired forms, such as pressure-overload induced cardiac hypertrophy and cardiac remodeling [19]. Recently, Song et al. reported that the possible mechanism by which atorvastatin functions in protecting against heart failure through downregulating ER stress response [20]. However, the effect of atorvastatin on PUMA-mediated myocardial apoptosis induced by AV-shunt and cyclic stretch is little known. Therefore, the purpose of this study was to test whether PUMA and myocardial apoptosis was induced by the AV shunt model in rat myocardium. Besides, we determined the molecular mechanism of PUMA regulation in cardiomyocytes apoptosis induced by cyclic stretch. We also used atorvastatin to inhibit the PUMA expression and apoptosis under AV shunt and cyclic stretch.

## Methods

### The aorta-caval shunt (AV shunt) rat model

AV shunt was performed on rats to induce volume overload. On the day of surgery, the Wistar rats weighing 280 to 330 g were anesthetized with 2% isoflurane and the vena cava and aorta were exposed via abdominal midline incision after confirming a fully anaesthetized state (e.g. no response to toe pinching). The aorta-caval shunt was produced as previously described [14]. The rats were euthanized with an overdose of isoflurane. All study protocols were approved by our Institutional Committee of Animal Care and Use (Protocol number #0990816001) and were carried out in accordance with the Guide for the Care and Use of Laboratory Animals (NIH publication No. 86–23, revised 1996).

### Cardiomyocytes culture

Cardiomyocytes were obtained from Wistar rats aged 2–3 days old by trypsinization, as previously described [21]. Cultured cardiomyocytes thus obtained were > 95% pure as revealed by observation of contractile characteristics with a light microscope and stained with anti-desmin antibody (Dako Cytomation, Glostrup, Denmark). Cardiomyocytes were seeded on flexible membranes base of six culture wells at a cell density of 5X10^5^ cells per well in Ham’s F-10 containing 20% fetal calf serum. After 3 days in culture, cells were transferred to serum-free medium (Ham’s F-10) and subjected to cyclic stretch.

### In vitro cyclic stretch on cultured cardiomyocytes

The Flexcell FX-2000 strain unit consists of a vacuum unit linked to a valve controlled by a computer program. Cardiomyocytes cultured on the flexible membrane base were subjected to cyclic stretch produced by this computer-controlled application of sinusoidal negative pressure with a peak level of ≅ 15 kPa at a frequency of 1 Hz (60 cycles per min) for various periods of time. To determine the roles of c-Jun N-terminal kinase (JNK), p38 or p44 mitogen-activated protein (MAP) kinase in the expression of stretch-induced PUMA expression, cardiomyocytes were pretreated with SP600125 (a potent, cell permeable, selective, and reversible inhibitor of JNK, 20 μM, Calbiochem, San Diego, CA), SB203580 (a highly specific, cell permeable inhibitor of p38 kinase, 3 μM, Calbiochem), PD98059 (a specific and potent inhibitor of ERK kinase, 50 μM, Calbiochem) or N-acetylcysteine (a free radical scavenger, 500 μM) for 30 min, respectively, followed by cyclic stretch.

### Western blot analysis

A Western blot was performed as previously described [14]. Goat monoclonal anti-PUMA antibody (1: 200; Santa Cruz Biotechnology, Santa Cruz) was used. Equal protein loading of the samples was verified by incubating with the monoclonal antibody α-tubulin.

### Reverse transcription-polymerase chain reaction (RT-PCR)

RT-PCR was performed as previously described [14]. The primers used were as follows: PUMA, 5’-d(CCCTGGAGGGTCCTGTACAA)-3’ (forward) and 5’-d(CTCTGTGGCCCCTGGGTAA)-3’ (reverse); and GAPDH, 5’-d(CATCACCATCTTCCAGGAGC) (forward) and 5’-d(GGATGATGTTCTGGGCTGCC)-3’ (reverse).

### Electrophoretic mobility shift assay (EMSA)

Nuclear protein concentrations from cultured cardiomyocytes were determined by Biorad protein assay. Consensus and control oligonucleotides (Santa Cruz Biotechnology) were labeled by polynucleotides kinase incorporation of [γ^32^-P]ATP. After the IRF-1 was radio-labeled, the nuclear extracts (4 μg of protein in 2 μl of nuclear extract) were mixed with 20 pmol of the appropriate [γ^32^-P]ATP-labeled consensus or mutant oligonucleotide in a total volume 20 μl for 30 min at room temperature. The samples were then resolved on a 4% polyacrylamide gel. Gels were dried and imaged by autoradiography. Controls were performed in each case with mutant or cold oligonucleotides to compete with labeled sequence.

### RNA interference

Cardiomyocytes were transfected with 800 ng siRNA of PUMA or JNK (Dharmacon, Lafayette, Colorado, USA). PUMA or JNK siRNA are target-specific 21 nt siRNAs according to a computer program provided by Dharmacon. The PUMA and JNK targeted base sequences were, as follows: sense: 5’-AAGAAGAGCAACAUCGACA and 5’-CGUGGAUUUAUGGUCUGUGdTdT-3’, respectively; and antisense: 5’-P. ACAAGAAGAGCAACAUCGA and 5’-CACAGACCAUAAAUCCACCdTdT-3, respectively. Green fluorescent protein (GFP) siRNA was used as a negative control, with the following: sense: 5’-P.GGCUACGUCCAGGAGCGCACC-3’ and antisense: 5’-P.UGCGCUCCUG GACGUAGCCUU-3’ (Dharmacon). After overnight incubation, cells were stretched and subjected to analysis by Western blot, EMSA, immunohistochemistry and detection of apoptosis.

### Promoter activity assay

A human PUMA promoter construct was generated as previously described (human PUMA: -2183 to −1490, containing an IRF-1 binding site [13]). The plasmids were transfected into cardiomyocytes using a low pressure-accelerated gene gun (Bioware Technologies, Taipei, Taiwan) essentially following the protocol from the manufacturer. Test plasmid (2 μg) and control plasmid (pGL4-Renilla luciferase; 0.02 μg) were co-transfected with a gene gun in each well, and then replaced with normal cultured medium. Following 6 hours of cyclic stretch, cell extracts were prepared for the Dual-Luciferase Reporter Assay System (Promega Corp., Madison, Wisconsin, USA) and measured for dual luciferase activity by luminometer (Turner Designs Inc., Sunnyvale, California, USA).

### Measurement of interferon-γ concentrations

Conditioned medium from cardiomyocytes subjected to cyclic stretch and those from control (unstretched) cells were collected for IFN-γ measurement. IFN-γ levels were measured using a quantitative sandwich enzyme immunoassay technique (R & D Systems, Minneapolis, MN, USA).

### Flow cytometric analysis for apoptotic quantification

Cardiomyocytes were fixed with 70% ethanol and treated with RNase. Then nuclei were stained with propidium iodide (Molecular Probes, Eugene, Oregon) and FITC Annexin V. DNA content was measured using by a FACS Calibur flow cytometer and Cell Quest software (Becton Dickinson, Franklin Lakes, NJ, USA). Ten thousand cells were counted in all assays. Apoptotic cells were quantified as the percentage of cells stained with Annexin V.

### Terminal deoxynucleotidyl transferase-mediated dUTP nick-end labeling (TUNEL) assay

DNA fragmentation was determined by TUNEL using the ApopTag peroxidase in situ apoptosis detection kit (Chemicon International, Temecula, CA, USA). TUNEL assay was performed as previously described [14]. Fluorescence signals were obtained with a confocal microscope (Nikon D-ECLIPSE) and assayed using its associated image processing and analysis software.

### Statistical analysis

All results were expressed as mean + S.E.M. Statistical significance was evaluated using analysis of variance (ANOVA; GraphPad Software Inc., San Diego, CA, USA). The Dunnett’s test was used to compare multiple groups to a single control group. The Turkey-Kramer comparison was used for pairwise comparisons between multiple groups following the ANOVA. A p < 0.05 was considered as significant.

## Results

### AV shunt enhances the expression of myocardial PUMA protein and mRNA expression

**Figure 1:**
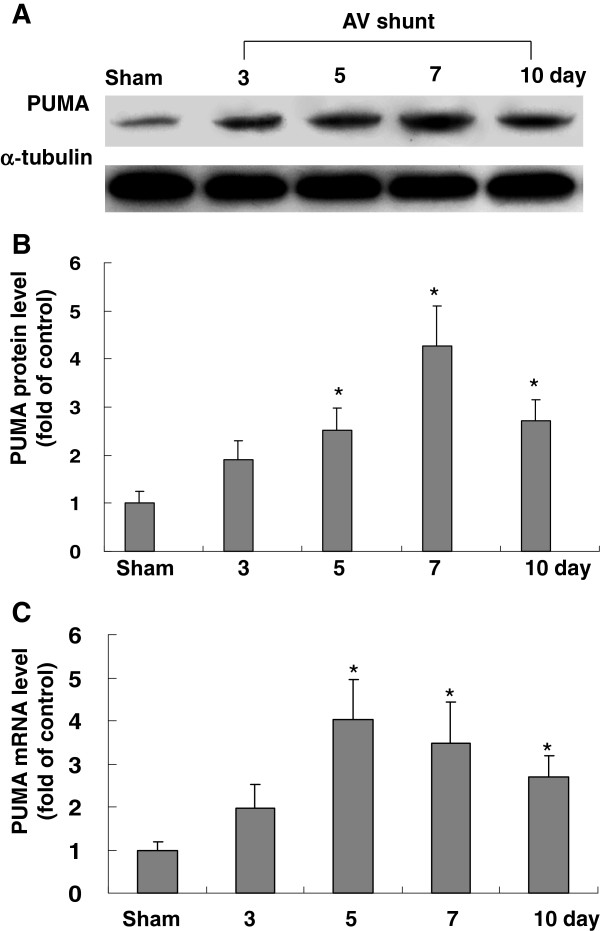
**Effect of in vivo model of aorta-caval shunt (AV shunt) on myocardial PUMA protein levels.** (**A**) Representative Western blots for PUMA in rat myocardium after short-term induction of AV shunt. (**B**) Quantitative analysis of PUMA protein levels. The values have been normalized to α-tubulin measurement and then expressed as a ratio of normalized values to PUMA protein in sham. (n = 5 per group). *P < 0.05 vs. control. ^+^P < 0.01 vs. control. (**C**) Fold increases in PUMA mRNA as a result of induction of AV shunt. The values from experiment groups have been normalized to match GADPH measurement and then expressed as a ratio of normalized values to mRNA in sham group. *P < 0.05 vs. sham group. (n = 5 per group).

As shown in Figure 1, the PUMA protein expression in rat myocardium significantly increased in rats with AV shunt for 7 days. Real-time PCR also showed that PUMA mRNA was up-regulated after AV shunt. These findings indicate that PUMA is induced by volume overload in rat myocardium.

### Atorvastatin reduces myocardial PUMA protein expression induced by AV shunt

**Figure 2:**
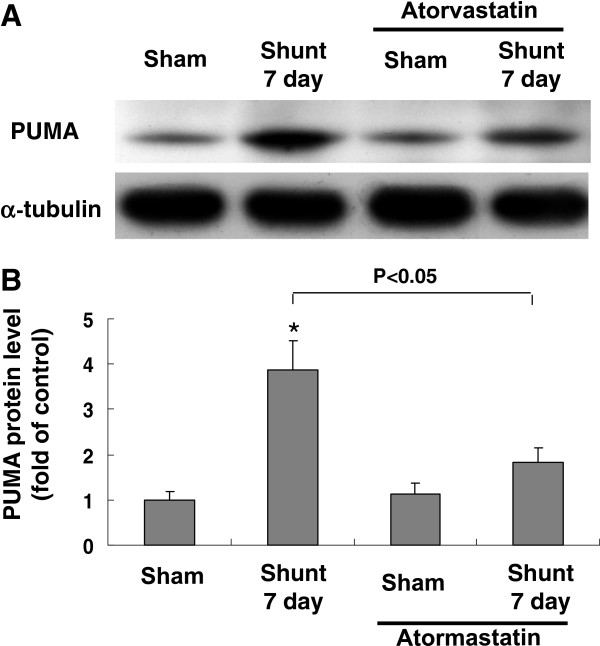
**Effects of atorvastatin on rat myocardium PUMA protein expression induced by AV shunt.** (**A**) Representative Western blots for PUMA after induction of AV shunt with or without treatment with atorvastatin. (**B**) Quantitative analysis of PUMA protein levels. The values from myocardium after AV shunt have been normalized to matched α-tubulin measurement and then expressed as a ratio of normalized values to protein in control group (n = 3 per group). *P < 0.01 vs. control.

The PUMA protein increased 3.6-fold at 7 days of AV shunt when compared with the sham group (Figure 2). Since most oral dose of statins used in animal study ranges from 10 to 50 mg/kg/day, we used 30 mg/kg/day in the present study. Treatment with atorvastatin at 30 mg/kg/ day orally for 7 days significantly blocked the increase of PUMA protein induced by AV shunt. However, treatment with atorvastatin in the sham group did not affect the protein expression of PUMA. These findings revealed that PUMA protein expression induced by AV shunt was reduced by atorvastatin. AV shunt for 10 days may induce congestive heart failure in rats and therefore the PUMA expression is changed as compared with 7 days.

### Atorvastatin inhibits apoptosis induced by AV shunt

**Figure 3:**
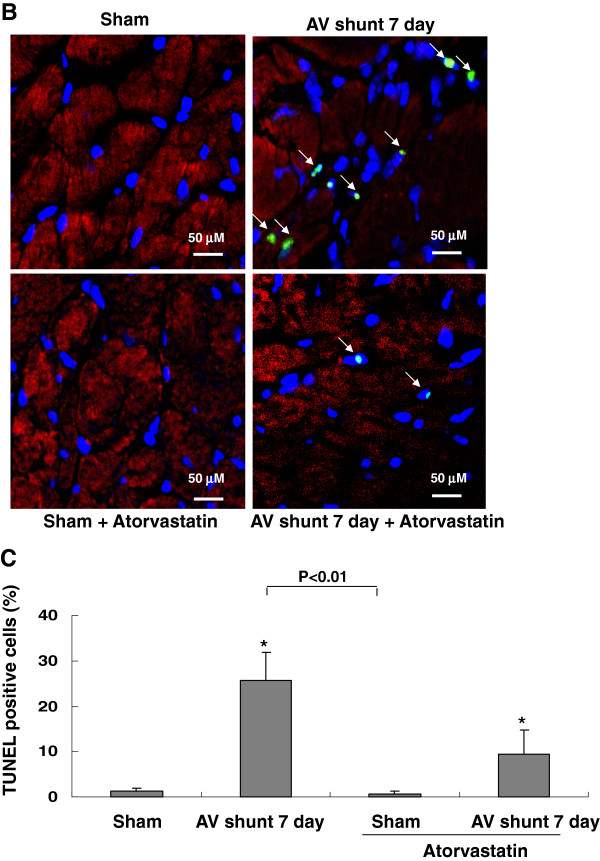
**AV shunt enhances rat myocardial apoptosis.** (**A**) Representative microscopy images of rat myocardium after AV shunt, treatment of atorvastatin before AV shunt then stained with a TUNEL kit. Arrow means TUNEL positive cells. Similar results were observed in another two independent experiments. (**B**) Quantitative analysis of TUNEL positive cells (%).*P < 0.01 vs. control.

As shown in Figure 3, AV shunt significantly increased apoptotic nuclei. Treatment with atorvastatin significantly reduced TUNEL positive nuclei. This result indicates that atorvastatin inhibits myocardial apoptosis induced by AV shunt.

### Cyclic stretch enhances PUMA protein and mRNA expression in cardiomyocytes

**Figure 4:**
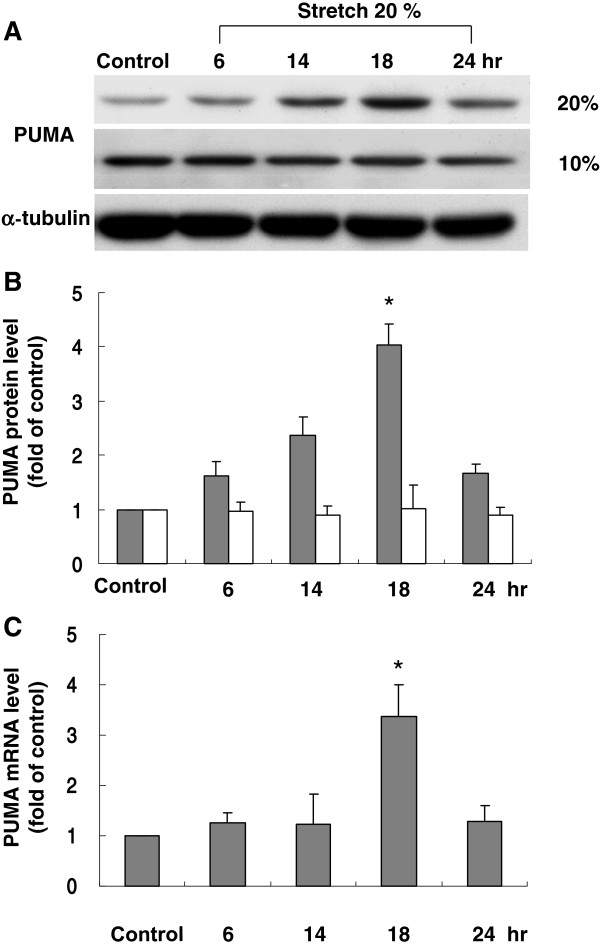
**Effects of cyclic stretch on PUMA expression in cardiomyocytes.** (**A**) Representative Western blots for PUMA in cardiomyocytes subjected to cyclic stretch by 20 or 10 % for various periods of time. (**B**) Quantitative analysis of PUMA protein levels. The values from stretched cardiomyocytes have been normalized to matched α-tubulin measurement and then expressed as a ratio of normalized values to protein in control group (n = 4 per group). *P < 0.01 vs. control. (**C**) Quantitative analysis of PUMA mRNA levels. The values from stretched cardiomyocytes have been normalized to matched GAPDH measurement and then expressed as a ratio of normalized values to mRNA in control group (n = 5 per group). *P < 0.01 vs. control.

The level of PUMA protein began to increase as early as 6 h after stretch to 20% elongation was applied, reached a maximum of 2.2-fold over the control by 14 h and remained elevated up to 18 h. When cardiomyocytes were stretched at 10% elongation, the level of PUMA protein was similar to that of control without stretch (Figure 4A4B). The real-time PCR showed that PUMA mRNA increased significantly after 14 h of stretch at 20% elongation (Figure 4C). These results revealed that PUMA expression in cardiomyocytes was induced by stretch. The effect of PUMA expression was maximal at 18 hours after stretch but not at 24 hours, indicating the transient effect of mechanical stretch on PUMA expression in cardiac myocytes.

### Stretch-induced PUMA protein expression in cardiomyocytes is mediated by JNK and IFN-γ

**Figure 5:**
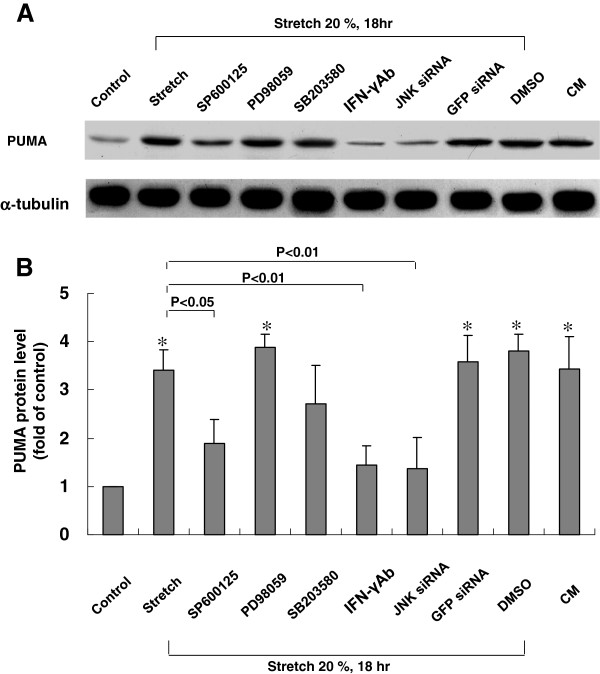
**Effects of MAP kinase inhibitors on PUMA protein expression induced by cyclic stretch in cardiomyocytes.** (**A**) Representative Western blots for PUMA protein levels in cardiomyocytes subjected to cyclic stretch in the absence or presence of MAP kinase inhibitors, IFN-γ Ab, siRNA and vehicle (DMSO 0.1 %). CM = conditioned medium. (**B**) Quantitative analysis of PUMA protein levels. The values from stretched cardiomyocytes have been normalized to matched α-tubulin measurement and then expressed as a ratio of normalized values to protein in control group (n = 4 per group). *P < 0.01 vs. control.

Cardiomyocytes were stretched by 20% for 18 h in the presence and absence of various inhibitors or siRNA to determine the signaling pathway mediating the stretch-induced increases in PUMA expression in cardiomyocytes. As shown in Figure 5, the stretch-induced increases of PUMA proteins were significantly blocked after the addition of SP600125 30 min before stretch. The PUMA protein induced by stretch was not affected by the addition of PD98059, but partially blocked by the addition of SB203580. Moreover, JNK siRNA also completely blocked the PUMA expression induced by cyclic stretch. Furthermore, conditioned medium alone also had a similar effect on PUMA expression levels as cyclic stretch, whereas DMSO alone, as a vehicle control, and control siRNA did not affect PUMA expression levels following cyclic stretch. Addition of IFN-γ monoclonal antibody 30 min before stretch also significantly blocked the expression of PUMA induced by cyclic stretch. These results indicate that PUMA protein expression induced by stretch in cardiomyocytes is mediated by JNK and IFN-γ.

### Cyclic stretch enhances IRF-1 binding activity

**Figure 6:**
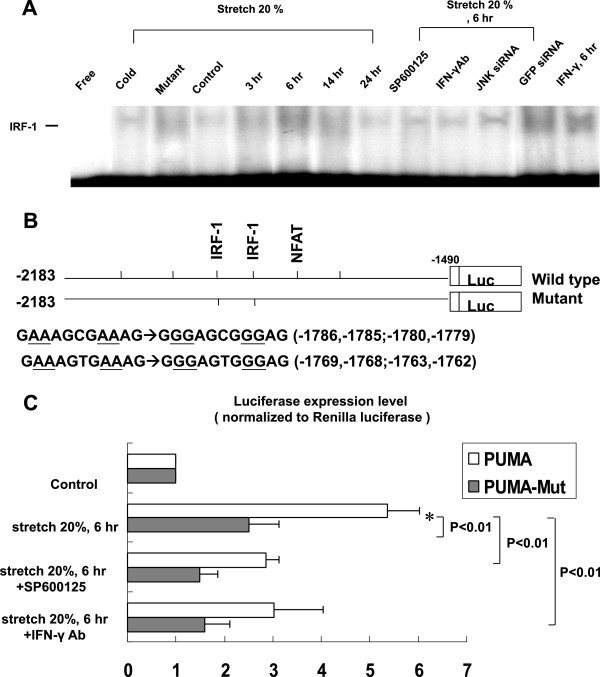
**Effects of cyclic stretch on PUMA binding and promoter activity in cardiomyocytes.** (**A**) Representative EMSA showing protein binding to IRF-1 oligonucleotide in nuclear extracts of cardiomyocytes following cyclic stretch during various times and in the absence or presence of JNK inhibitors or siRNA, IFN-γ antibody or addition of 200 pg/ml IFN-γ. Arrows indicate the mobility of the complex. Similar results were found in another two independent experiments. Cold oligo means unlabeled IRF-1 oligonucleotide. (**B**) Constructs of PUMA promoter gene. (**C**) Quantitative analysis of PUMA promoter activity. Cardiomyocytes were transiently transfected with pPUMA-Luc by a gene gun. The luciferase activity in cell lysates was measured and was normalized with Renilla activity (n = 3 per group). *P < 0.01 vs. control.

Cyclic stretch significantly began to increase the DNA-protein binding activity of IRF-1 in cardiomyocytes at 3 h after stretch and reached a maximum at 6 h and remained elevated for 24 h (Figure 6A). An excess of unlabeled IRF-1 oligonucleotide competed with the probe for binding IRF-1 protein, whereas an oligonucleotide containing a 2-bp substitution in the IRF-1 binding site did not compete for binding. Addition of SP600125, JNK siRNA and IFN-γ antibody (5 mg/mL, purchased from R&D Systems) 30 min prior to stretch abolished the DNA-protein binding activity induced by cyclic stretch. Moreover, exogenous IFN-γ also induced IRF-1 binding activity. These results demonstrated that stretch enhanced IRF-1 binding activity was mediated by IFN-γ and JNK in cardiomyocytes.

### Cyclic stretch increases PUMA promoter activity through IRF-1 in cardiomyocytes

The PUMA promoter construct contains CREB, NFAT, NF-κB and IRF-1 binding sites (Figure 6B). As shown in Figure 6C, cyclic stretch for 6 h significantly activated IRF-1 promoter. This result indicates that PUMA expression is induced at transcriptional level during stretch in cardiomyocytes. Besides, transient transfection of PUMA-Mut plasmid and addition of SP600125 and IFN-γ Ab abolished the promoter activity induced by stretch.

### Atorvastatin reduces PUMA expression and apoptosis induced by cyclic stretch in cardiomyocytes

**Figure 7:**
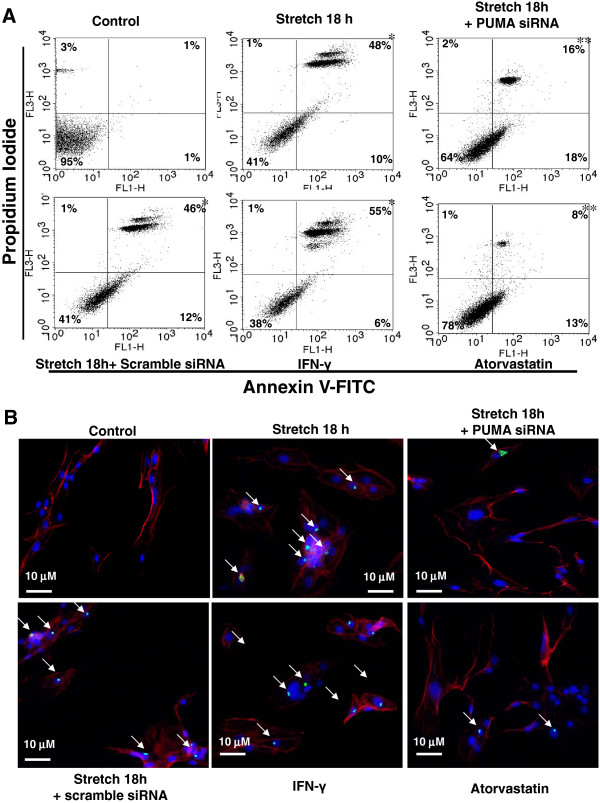
**Effect of PUMA on stretch-induced apoptosis in cardiomyocytes.** (**A**) Cardiomyocytes were subjected to cyclic stretch for 18 h, addition of 10 μM atorvastatin, siRNA before stretch or addition of 200 pg/ml IFN-γ alone. Quantification of the apoptotic fractions was performed using FACScan. Cells that stain negative for both Annexin V and PI are alive. Cells that stain positive for Annexin V and negative for PI are undergoing apoptosis. Cells that stain positive for both Annexin V and PI are in the end stage of apoptosis called second apoptosis. (n = 3). *P < 0.05 vs. control. **P < 0.05 vs. stretch 18 h. (**B**) Representative microscopy images of cardiomyocytes after cyclic stretch for 18 h, addition of atorvastatin, siRNA before stretch or addition of IFN-γ, then stained with a TUNEL kit. Similar results were observed in another two independent experiments. Arrow indicates TUNEL positive cells.

As shown in Additional file 1: Figure S1, atorvastatin significantly reduced PUMA protein expression induced by stretch. As PUMA is an apoptosis related gene and enhanced by volume overload, we speculated that PUMA is involved in apoptosis during cyclic stretch. As shown in Figure 7A, apoptosis was assessed by PI/annexin V double staining and FACS analysis. The percentage of cells stained with annexin V was elevated following stretch for 18 h and the addition of IFN-γ. These increases of annexin V positive cells induced by stretch were significantly reversed by PUMA siRNA and 10 μM atorvastatin. Besides, a significant increase in TUNEL-positive nuclei was present after stretch for 18 h and the addition of IFN-γ (Figure 7B). These increases in TUNEL-positive nuclei of cardiomyocytes induced by stretch were significantly reversed by PUMA siRNA and atorvastatin. These findings demonstrate that PUMA mediates stretch-induced apoptosis of cardiomyocytes is inhibited by atorvastatin.

### Cyclic stretch stimulates the secretion of IFN-γ from cultured cardiomyocytes

As shown in Additional file 1: Figure S2, cyclic stretch significantly increased the IFN-γ secretion from cultured cardiomyocytes at 2 h, and it remained elevated for 24 h. This result indicates that stretch stimulates the secretion of IFN-γ from cultured cardiomyocytes.

## Discussion

In the present study, we demonstrated that (1) in vivo acute hemodynamic overload enhances rat myocardium PUMA expression, (2) atorvastatin inhibits myocardial PUMA expression and apoptosis induced by AV shunt, (3) cyclic stretch upregulates PUMA expression in rat cardiomyocytes, (4) JNK MAP kinase and the IRF-1 transcription factor are involved in the signaling pathway of PUMA induced by stretch in cardiomyocytes, (5) IFN-γ mediates the increase in PUMA expression induced by cyclic stretch, (6) cyclic stretch induces cardiomyocytes apoptosis via PUMA, (7) cardiomyocytes apoptosis induced by cyclic stretch is inhibited by atorvastatin.

We demonstrated that myocardium PUMA protein and mRNA expression were up-regulated in a model of AV shunt in myocardium. We have previously demonstrated that aortic PUMA expression was induced by both pressure and volume overload [14]. In this study, we presented that PUMA expression was induced by cyclic stretch in cardiomyocytes. Cyclic stretch of cardiomyocytes increased both the expression of PUMA protein and mRNA. PUMA protein expression was up-regulated in both a time- and load- dependent manner by cyclic stretch. It has been demonstrated that PUMA expression is induced under various stress in cancer, neuronal and cardiovascular cells [12]. Besides, Li et al. also reported that hypoxia/reoxygenation could upregulate the expression of PUMA in cardiomyocytes [22]. Nickson et al. have shown that ER-stress induced by thapsigargin or tunicamycin leads to transcriptional upregulation of PUMA in rat neonatal cardiac myocytes [16]. In the present study, we performed in vivo model of AV shunt in adult rats and performed in vitro stretch experiment with neonatal cardiomyocytes. AV shunt model is difficult to perform in neonatal rats. The mechanism of stretch-induced apoptosis in neonatal cardiomyocytes may not apply to the findings in the in vivo AV shunt model in adult animal.

Our findings indicated that myocardium apoptosis was up-regulated in a model of volume overload. It has been suggested that BAX is upregulated and may play a role in cardiomyocyte apoptosis in heart failure due to volume overload [23]. Besides, Dent et al. also reported that upregulation of BAX may play a major role in cardiomyocyte apoptosis in heart failure due to volume overload in male rats [24]. According to these studies, we may suggest that volume overload enhance apoptosis of cardiomyocytes. Besides, we also found that atorvastatin inhibited PUMA expression and apoptosis induced by AV-shunt. Functional inhibition of PUMA has been indicated a new potential therapeutic target for inhibiting the progression of hypertrophy to heart failure [25]. Previous study has reported that atorvastatin significantly improves cardiac function after myocardial infarction due to a decrease in myocardial apoptosis [17]. Besides, it has been shown that treatment with atorvastatin reversed established cardiac hypertrophy and interstitial fibrosis and improved cardiac function [26]. Wu et al. also suggested that the administration of valsartan can ameliorate the ER stress through blocking the CHOP/PUMA-mediated myocardial apoptosis in streptozotocin-induced diabetic rats [27]. Moreover, deletion of PUMA appears to protect hematopoietic stem cells during ionizing radiation for malignancy [28]. Accordingly, we may suggest that PUMA inhibitor could be a potential candidate to improve the myocardial apoptosis and heart failure.

We found that cyclic stretch enhanced IFN-γ expression in cardiomyocytes (Additional file 1: Figure S2 ). Our results also suggested that IFN-γ is responsible for IRF-1-DNA binding in cardiomyocytes. We further demonstrated that the JNK1 inhibitor and siRNA significantly inhibited PUMA expression induced by stretch. These results indicated that the JNK MAP kinase pathway is the major pathway involved in the induction of PUMA by stretch and mediates the increased binding activity of IRF-1 in cardiomyocytes. Zhao et al. suggested that the JNK-potentiated Akt-FoxO3a and JNK-mediated c-Jun pathways cooperatively trigger PUMA expression in ovarian cancer cells [29]. Besides, our reporter gene assay found that increased transcriptional activity of PUMA promoter by stretch was IRF-1 dependent. In general, PUMA is transactivated by p53 under various stresses, like DNA damage, hypoxia and ER stress. However, PUMA is also activated by IRF-1 to begin p53-independent apoptotic responses to nongenotoxic stimuli, including growth factor/cytokine deprivation, ischemia/reperfusion and ER stress [13]. Otherwise, CHOP could also transcriptional regulates PUMA expression. Kumar et al. reported that IRF-1 may be involved in the pathological response of human fetal myocyte to septic serum from patients. Besides, their study also indicated that c-Jun NH2-terminal kinase may play an important role in the induction of fetal myocyte apoptosis by addition of human septic serum [30]. It has also been demonstrated that p53 activation plays a crucial role in PUMA-mediated ROS generation induced by silibinin in A431 cells [31]. In this study, our finding indicated that JNK and IRF-1 may involve in the PUMA expression induced by stretch in cardiomyocytes. Furthermore, we found that an exogenous addition of IFN-γ to non-stretched cardiomyocytes was sufficient to induce cardiomyocytes apoptosis. Chae et al. also reported that treatment of neonatal rat ventricular cardiomyocytes with IFN-γ induces apoptosis via an NO-dependent pathway [32]. However, it has been indicated that incubation of cardiomyocytes with TNF-α, but not with IFN-γ, caused significant caspase-3 activation and apoptosis [33]. Li et al. [33] used adult mice cardiomyocytes, whereas Chae et al. [32] and our studies used neonatal rat cardiomyocytes. Different species may explain the discrepancy.

In this study, we demonstrated that cardiomyocytes apoptosis induced by stretch is mediated by PUMA. It has been demonstrated that cyclic stretch could induce cardiovascular cells apoptosis [34]. Previously, our group has also demonstrated cyclic stretch could induce cardiovascular cells apoptosis [21]. Recent studies showed that ER stress response could also cause cardiomyocytes apoptosis during the progression of heart failure [35]. Li et al. also revealed that PUMA participates in hypoxia/reoxygenation-triggered cardiomyocyte apoptosis by interfering with mitochondrial pathway [36]. PUMA has been reported as an essential mediator of cardiomyocyte death upon ischemia/reperfusion injury [12]. These observations are consistent with our data regarding PUMA-mediated cardiomyocytes apoptosis. Besides, PUMA also plays an important role on apoptosis in other cells. Antony et al. implicate that Bim-independent apoptosis by BITC in cancer cells is mediated by PUMA [37]. It has also been reported that in CCHFV-infected hepatocytes the over-expression of PUMA, Noxa and CHOP may involve in the crosstalk between the ER-stress and mitochondrial apoptosis [38].

## Conclusions

In summary, our result reveals that atorvastatin inhibits myocardium PUMA expression and apoptosis induced by AV shunt. Cyclic stretch enhances PUMA expression in cardiomyocytes. The stretch-induced PUMA is mediated by IFN-γ, JNK MAP kinase and IRF-1 pathway. We also demonstrate that atorvastatin reduces the PUMA-mediated apoptosis induced by stretch. Further studies can help to identify the mechanism through which PUMA mediates apoptosis and to clarify the specific role of PUMA in heart failure.

## Abbreviations

AV shunt, Aorta-caval shunt; HMG-CoA, 3-hydroxy-3-methylglutaryl coenzyme A; JNK, c-Jun N-terminal kinase; ER, Endoplasmic reticulum; EMSA, Electrophoretic mobility shift assay; ERK1/2, Extracellular signal-regulated kinase 1/2; IRF-1, Interferon regulator factor-1; INFγ, Interferon-γ; MAP, Mitogen-activated protein; NAC, N-acetylcysteine; PUMA, p53-upregulated modulator of apoptosis; RT-PCR, Reverse transcription-polymerase chain reaction; siRNA, Small interfering RNA; TUNEL, Terminal deoxynucleotidyl transferase-mediated dUTP nick-end labeling; UPR, Unfolded protein response.

## Competing interests

The author(s) declare that they have no competing interests.

## Authors’ contribution

Wen-Pin Cheng has participated in the design of the study and drafted the manuscript. Gong-Jhe Wu has made substantial contributions to conception and design, or acquisition of data, or analysis and interpretation of data. Bao-Wei Wang has made substantial contributions to conception and design, or acquisition of data, or analysis and interpretation of data. Kou-Gi Shyu has given final approval of the version to be published. All authors read and approved the final manuscript.

## Supplementary Material

Additional file 1 **Figure S1 and S2.Figure S1.** Effect of atorvastatin on PUMA expression induced by stretch in cardiomyocytes. (A) Representative Western blots for PUMA protein levels in cardiomyocytes subjected to cyclic stretch in the absence or presence of 10 μM atorvastatin. (B) Quantitative analysis of PUMA protein levels. The values from stretched cardiomyocytes have been normalized to matched α-tubulin measurement and then expressed as a ratio of normalized values to protein in control group (n = 3 per group). *P < 0.01 vs. control. **Figure S2:** Cyclic stretch increases release of rat IFN-γ from cardiomyocytes subjected to cyclic stretch by 20% for various periods of time (n = 3 per group). *P < 0.01 vs. control. Click here for file
