# Supplementary material for: Regulation of PUMA induced by mechanical stress in rat cardiomyocytes
Source: J Biomed Sci. 2012 Aug 3;19(1):72. doi: 10.1186/1423-0127-19-72 (PMC3430577; doi:10.1186/1423-0127-19-72)
Supplement: Additional file 1 — Figure S1 and S2.Figure S1. Effect of atorvastatin on PUMA expression induced by stretch in cardiomyocytes. (A) Representative Western blots for PUMA protein levels in cardiomyocytes subjected to cyclic stretch in the absence or presence of 10 μM atorvastatin. (B) Quantitative analysis of PUMA protein levels. The values from stretched cardiomyocytes have been normalized to matched α-tubulin measurement and then expressed as a ratio of normalized values to protein in control group (n = 3 per group). *P < 0.01 vs. control. Figure S2: Cyclic stretch increases release of rat IFN-γ from cardiomyocytes subjected to cyclic stretch by 20% for various periods of time (n = 3 per group). *P < 0.01 vs. control. [file 1423-0127-19-72-S1.doc]

**Supplemental Figure legends**

**Figure I:** Effect of atorvastatin on PUMA expression induced by stretch in cardiomyocytes. (A) Representative Western blots for PUMA protein levels in cardiomyocytes subjected to cyclic stretch in the absence or presence of 10 μM atorvastatin. (B) Quantitative analysis of PUMA protein levels. The values from stretched cardiomyocytes have been normalized to matched α-tubulin measurement and then expressed as a ratio of normalized values to protein in control group (n=3 per group). *P<0.01 vs. control.

**Figure II:** Cyclic stretch increases release of rat IFN-γ from cardiomyocytes subjected to cyclic stretch by 20% for various periods of time (n = 3 per group). *P<0.01 vs. control.
